# Exploring perceptions of group antenatal Care in Urban India: results of a feasibility study

**DOI:** 10.1186/s12978-018-0498-3

**Published:** 2018-04-03

**Authors:** R. Rima Jolivet, Bella Vasant Uttekar, Meaghan O’Connor, Kanchan Lakhwani, Jigyasa Sharma, Mary Nell Wegner

**Affiliations:** 1 Maternal Health Task Force, Women & Health Initiative, Department of Global Health and Population, Harvard T.H. Chan School of Public Health, 651 Huntington Avenue, Boston, MA 02115 USA; 2 Centre for Operations Research and Training, 402 Woodland Apartment, Race Course Circle, Vadodara, Gujarat 390 007 India; 3 Maternal Health Task Force, Women & Health Initiative, Harvard T.H. Chan School of Public Health, 651 Huntington Avenue, Boston, MA 02115 USA; 4 Department of Global Health and Population, Harvard T. H. Chan School of Public Health, 677 Huntington Ave, Boston, MA 02115 USA

**Keywords:** Antenatal care, Prenatal care, Group care, Feasibility study, Qualitative methods, India

## Abstract

**Background:**

Making high-quality health care available to all women during pregnancy is a critical strategy for improving perinatal outcomes for mothers and babies everywhere. Research from high-income countries suggests that antenatal care delivered in a group may be an effective way to improve the provision, experiences, and outcomes of care for pregnant women and newborns. A number of researchers and programmers are adapting group antenatal care (ANC) models for use in low- and middle-income countries (LMIC), but the evidence base from these settings is limited and no studies to date have assessed the feasibility and acceptability of group ANC in India.

**Methods:**

We adapted a “generic” model of group antenatal care developed through a systematic scoping review of the existing evidence on group ANC in LMICs for use in an urban setting in India, after looking at local, national and global guidelines to tailor the model content. We demonstrated one session of the model to physicians, auxiliary nurse midwives, administrators, pregnant women, and support persons from three different types of health facilities in Vadodara, India and used qualitative methods to gather and analyze feedback from participants on the perceived feasibility and acceptability of the model.

**Results:**

Providers and recipients of care expressed support and enthusiasm for the model and offered specific feedback on its components: physical assessment, active learning, and social support. In general, after witnessing a demonstration of the model, both groups of participants—providers and beneficiaries—saw group ANC as a vehicle for delivering more comprehensive ANC services, improving experiences of care, empowering women to become more active partners and participants in their care, and potentially addressing some current health system challenges.

**Conclusion:**

This study suggests that introducing group ANC would be feasible and acceptable to stakeholders from various care delivery settings, including an urban primary health clinic, a community-based mother and child health center, and a private hospital, in urban India.

## Plain English summary

Research from high-income countries suggests women who receive antenatal care (ANC) in a group may have better experiences and outcomes of care than those who get ANC through individual visits with a health care provider. However, there are few published studies on group ANC in low- and middle-income countries. In this study, we asked participants to share their views about whether group ANC could work and whether it would be acceptable in Vadodara, India. To conduct the study, we developed a “generic” model of group ANC, and then made sure the content matched the care guidelines from the Indian Ministry of Health and Family Welfare. We demonstrated one session of the group model to doctors, auxiliary nurse midwives, pregnant women, and support persons (mothers-in-law, mothers, husbands). These demonstrations were held in three different locations in Vadodara where ANC is typically provided: a private maternity hospital, a public health clinic, and a community-based mother and child health center. Focus group discussions, interviews, and a survey were used to learn from the participants what they thought about the group model. The feedback received through these methods showed that pregnant women and their families, as well as health care providers and administrators, thought it would be feasible and acceptable to conduct group ANC in Vadodara. Participants suggested ideas to better the chances for success in providing group ANC in this setting, including making sure that there is enough staff and space to support the model.

## Background

Making high-quality health care available to all women during pregnancy is a critical strategy for improving perinatal outcomes for mothers and babies everywhere. A recent conceptual framework published by the World Health Organization (WHO) includes provision of appropriate services, assurance of positive experience of care, and effective care delivery by the health system within one comprehensive definition of quality of maternal and newborn care in facilities. The framework emphasizes the importance of three types of outcomes of high quality care: coverage of key practices, health outcomes, and people-centered outcomes. It also calls for innovations in healthcare delivery to meet achieve these outcomes [1].

### Group Care in High-income Countries

Many studies from high-income countries have reported positive pregnancy experiences and improved birth outcomes among women receiving antenatal care (ANC) in a group, compared to those receiving traditional one-to-one ANC. In high-income settings, the most widespread model of group ANC with the most extensive evidence base is CenteringPregnancy® which combines clinical care, education, and peer support in one bundled program [2]. A randomized controlled trial of group care in the United States by Ickovics et al. [3] found a 33% decrease in the odds of preterm delivery for women in group care as well as better utilization of ANC, increased prenatal knowledge, increased readiness for labor and delivery, higher satisfaction with care, and higher rates of breastfeeding initiation than women receiving individual ANC. The study also found that there were no differences in the cost of group ANC compared to usual care [3]. Similarly, Earnshaw et al. [4] analyzed data from two separate randomized controlled trials of group ANC and reported that young, urban women in group ANC demonstrated higher levels of engagement and attended more ANC visits than controls. The results of a cluster-randomized controlled trial conducted in 14 urban health centers in the United States demonstrated that women in group ANC delivered significantly fewer infants who were small for gestational age compared to women in traditional ANC [5]. Studies have also demonstrated a significant improvement in appropriate pregnancy weight gain for women in group care compared to individual ANC [6, 7]. However, a Cochrane systematic review of four randomized controlled trials (two in the United States, one in Sweden, and one in Iran) (2015) found no differences in health outcomes between women in group and individual ANC, noting that the review was limited by the small number of trials and participants [8]. The overall positive outcomes reported by group ANC studies conducted in high-income settings suggest that, with some adaptations in the number and content of sessions to meet local guidelines, the group care format might improve quality and experiences of ANC for women in low- and middle-income countries (LMICs) as well.

### Group Care in low- and Middle-Income Countries

Although several researchers and programmers are currently exploring the implementation and outcomes of group ANC in LMICs, few studies have been published to date and the body of knowledge on the safety and effectiveness of group ANC in LMICs is limited [9]. Nevertheless, a few small, descriptive studies have assessed perceptions of the acceptability and feasibility of group care among various target audiences in LMICs. Patil et al. [10] assessed the feasibility and acceptability of group ANC with health care providers, decision makers, and women in Malawi and Tanzania, and reported positive responses. Ghani [11] surveyed frontline health workers in Egypt; responses indicated health workers felt the group model would have positive effects on early prevention and detection of complications, patient empowerment, and self-care behaviors, but could face barriers to implementation related to staff training requirements and cost. Arnold et al. [12] surveyed seven male partners of pregnant women who participated in group ANC in Botswana and reported positive responses such as satisfaction with care, increased access to information, and an experience of group care as respectful and sufficiently private.

The few research studies conducted to date that have evaluated the effects of group ANC in LMICs generally report positive results. In Iran, Jafari et al. [13] conducted a cluster-randomized trial of group ANC at 14 health care facilities with a total of 628 participants, 320 of whom received group ANC. The intervention resulted in reductions in the number of low birth-weight babies and preterm births, intrauterine growth restriction and perinatal loss, although none of these findings was statistically significant. Women receiving group care were, however, significantly more likely to take prenatal vitamins and iron, and to be using a contraceptive method at 2 months postpartum. Women receiving group ANC also reported significantly higher satisfaction with the information they received, the relationship with their provider, and the quality of care and service delivery, and scored significantly higher on the Kotelchuck Index assessing adequacy of ANC utilization [13, 14]. The positive outcomes of group ANC reported across settings provide sufficient rationale to explore the potential for this innovation in additional LMIC settings.

This paper describes the results of a qualitative research study conducted in Vadodara, India to explore the feasibility and acceptability of a model of group ANC designed for use in LMICs. By collecting the perspectives of care providers, care managers, and care recipients (pregnant women and their accompanying family members) on the strengths and challenges related to introducing an alternative, evidence-based group model of ANC, we aimed to answer two questions:

1. What are the likely strengths and challenges of implementing an evidence-based model of group ANC in the context of three types of care-delivery settings (private, public, and community-based) in urban India?
2. Are there any context-specific modifications or adaptations that would increase the potential of this model of ANC to: a) be implemented effectively within the local health system; and b) improve patient and provider experiences by responding to specific population health needs or health system problems in Vadodara?

## Methods

### Study setting and design

This research was conducted in Vadodara, a city located in the western Indian state of Gujarat, with a population of 2.3 million [15]. In Vadodara, pregnant women may receive ANC at a public, district tertiary-care hospital, sub-district hospitals, urban public health clinics (UPHCs), community-based maternal and child health centers (*anganwadis*), and private maternity hospitals. To assess stakeholder perceptions of group ANC within the unique patient populations served by these different types of facilities, we collected data in three settings: a private hospital, a UPHC, and a community-based *anganwadi*. The specific study sites were selected in collaboration with a local representative of the Indian Ministry of Health and Family Welfare (MOHFW), and permission was gained from physicians/administrators in those facilities. The study was carried out collaboratively by a team of researchers from the Centre for Operations Research and Training in Vadodara, and the Harvard T.H. Chan School of Public Health in Boston, MA.

We used a descriptive, qualitative study design to assess the feasibility and acceptability of group ANC to potential stakeholders. Given the novelty of the group ANC model in the study setting, we incorporated a demonstration of one mock session of group ANC into the study design to allow participants to visualize and directly experience the proposed innovation. The demonstration in each setting was followed immediately by data collection using qualitative methods, which are detailed below.

### Model

We conducted a systematic review of the published literature and a series of key informant interviews to gather evidence on the components of group antenatal care models in current use in LMICs. Two existing group models emerged as most influential in LMIC settings: nine out of the nineteen unique models identified reportedly drew from CenteringPregnancy® [2] and four out of nineteen reported they were informed by the Home Based Lifesaving Skills (HBLSS) program [16]. We synthesized the data collected through this systematic scoping review and compiled the common elements of the group ANC models reviewed to describe a “generic” model of group ANC specifically for use in LMICs. This evidence review and synthesis is described in Sharma, et al. [9].

We then reviewed the ANC guidelines from the MOHFW and added clinical and educational content to the “generic” model to ensure compliance with the recommended standards of care in the study setting [17, 18]. Two adaptations were made:

1. The number of group sessions was set at four (not inclusive of the initial intake visit): three antenatal sessions and one post-natal care (PNC) session. This number fulfills the Indian MOHFW’s minimum recommendation for four antenatal care visits (one intake visit plus three group antenatal visits) and includes the recommended PNC visit at 6 weeks postpartum.
2. The content for each of the four group sessions reflects the clinical care standards found in the most recent MOHFW maternal and newborn health register and guidelines for ANC and skilled attendance at birth.

### Participants

Study participants were recruited from two groups of key stakeholders: care receivers and care providers. Care receivers included pregnant women and any support person (e.g. mother, mother-in-law, and husband) whom the women chose to accompany them. Care providers included clinicians who provide ANC (physicians and auxiliary nurse midwives) (ANMs) from each of the study sites and administrators who coordinate the provision of care at these sites. Pregnant women age 18 or older, up to 26 weeks gestation, receiving antenatal care at the study site, and who had attended at least one previous ANC visit during the current pregnancy were eligible to participate. Health care providers were eligible if they were a nurse, midwife, physician, or physician-administrator who was working at the study site and involved in the provision of antenatal care (In the Indian health system, attending physicians often also serve as facility administrators). Physicians and auxiliary nurse midwives from each site were recruited directly by the study team. Pregnant women were recruited by staff at the three study sites using convenience sampling. Each participant was given lunch and compensation for their transportation costs (200 Indian rupees, which is equivalent to approximately $3 US dollars).

### Demonstration of the model

We developed an agenda for one mock session of group ANC based on our generic model components tailored to the local context, and used it to demonstrate the model for study participants. The demonstration for the care providers was conducted in English by two members of the Harvard research team (RJ and MO’C) who played the role of group facilitators, with translation into Gujarati from the CORT research team (BU and KL) as needed. A local physician and ANM were recruited to play the roles of the group facilitators during the demonstrations that were conducted for pregnant women and their support persons. These sessions were conducted in the local language (either Gujarati or Hindi) to maximize participants’ understanding and participation.

A total of four demonstrations of the group ANC model were conducted: one for providers/administrators (held at a central location), and one at each of the three study sites for women and their family members. Before each demonstration, the study team provided a short introduction to the research study and its aims, requested and collected signed consent, briefly outlined the group ANC model, and clarified that the participants would not be receiving actual clinical care. In the demonstration that was staged for care providers and administrators, participants were asked to play the role of pregnant women so they could observe and experience how care is provided in the group ANC model. The content and format for all four demonstrations was the same and is described in detail in Table 1. During the first 30 min, participants took and recorded their own weight and blood pressure, completed two worksheets about their diet and discomforts during this pregnancy, and saw the clinician in rotation for a brief (mock) physical exam. The co-facilitator opened the group with a short icebreaker. The icebreaker was followed by a facilitated discussion during which participants developed and agreed on shared behavioral norms and guidelines for the group. The rest of the session focused on the interactive learning component of the model: the participants engaged in a facilitated discussion about nutrition during pregnancy and participated in a group activity and discussion of common discomforts and danger signs during pregnancy. The co-leaders guided the discussions and activities using a facilitative leadership style to encourage active participation from the women. The demonstration ended with a short activity in which each participant shared one thing they learned, designed to summarize and close the session.

**Table 1:** Agenda for Group ANC Session Demonstration

Time	Topic	Leader	Props/Materials	Content
30	Physical self-assessment Psychosocial self-assessment	Co-leader 2 (auxiliary nurse midwife)	1. Digital scale 2. Portable blood pressure cuff 3. Mock flow chart 4. Nutrition worksheet 5. Common discomforts worksheet	• Show each participant how to take their weight and blood pressure and record the results on a mock flow chart. • Distribute two psychosocial self-assessment sheets and provide guidance on how to complete them.
	Provider physical assessment	Co-leader 1 (physician)	1. 1 charpoy and stool 2. 1 Privacy screen or curtain 3. Measuring tape 4. Fetoscope or Doppler 5. Mock chart	• Welcome women individually to the private corner where the clinical exam will take place. • Conduct a mock physical exam with each woman. • Ascertain that privacy and confidentiality are maintained.
10	Opening ice-breaker	Co-leader 2	Soft ball	• Explain the game whose aim is for everyone to introduce themselves and learn the names of others in the group. • Lead the game; help ensure everyone participates.
10	Group guidelines	Co-leader1 Co-leader 2	1. Flip chart or white board 2. Markers	• Facilitate a discussion to help the group develop its own guidelines for group conduct that is comfortable for everyone. Help the group brainstorm, offering probes. • Ask a volunteer to write the group’s agreements on a flip chart, and tell the group that these guidelines will be on display during all their group sessions. • Tell the group that all members and any visitors will be asked to sign a confidentiality agreement.
30	Group discussion	Co-leader 1 Co-leader 2	Nutrition worksheet	• Use the worksheet to lead a facilitated discussion on nutrition. • Offer discussion prompts, e.g. “Does anyone know which of the foods here are high in calcium? Why is calcium important in pregnancy? What can you do if you don’t like any of those foods?” • Acknowledge each participant’s comment or question, refer back to the group for further reflection and discussion, then summarize or expand on the discussion. • Once the topic has been exhausted or it is time to move on, wrap up the discussion, ensuring that participants’ questions have been answered satisfactorily.
10	Group learning activity	Co-leader 1 Co-leader 2	1. Common discomforts worksheet 2. Green light/red light paddles	• Use the green light/red light paddles to stimulate group discussion about common discomforts during pregnancy, share ways to address them, and discuss when a symptom should be considered a danger sign.
10	Closing ritual/activity	Co-leader 2 Co-leader 1	Yarn ball and scissors	• Explain the closing ritual designed to encourage participants to recap what they learned in the group, illustrate the connections that were made, and end the session.

### Data collection

Data was collected through a mix of focus group discussions, in-depth interviews, and a written survey. The questions used to guide the focus group discussions and in-depth interviews were designed to elicit participants’ perceptions regarding the feasibility and acceptability of the group ANC model in general, as well as specific elements of the model, and to capture any perceived barriers to implementation along with recommendations for how to overcome them. All physicians participated in an in-depth interview. All ANMs participated in a focus group discussion followed by an individual survey that asked the same questions as those posed in the in-depth interview. The purpose of the survey was to validate the focus group discussion findings and check for any cognitive bias, i.e., groupthink, which may have occurred during the focus group discussion. The pregnant women and their support persons were randomly assigned to either a focus group discussion or an in-depth interview. The only non-random assignments were to ensure that women and their support persons were not assigned to the same focus group discussion; this separation was made to avoid courtesy and other biases resulting from any potential power imbalances between women and their support persons or other factors. All focus group discussions and in-depth interviews were recorded and transcribed in Gujarati, and then translated into English. English translations were reviewed to identify themes, coded, and analyzed using Atlas.ti, version 8 software (Berlin, Germany, 2017). Themes were extracted from the participants’ responses to each question in the focus group discussion, in-depth interview, and survey, and are presented below in the order they were asked. Codes were derived from the research questions and emerged from the collected data. They were color-coded and categorized into themes, and illustrative quotes were selected to represent each emergent theme. Some further quotes were selected to add in-depth understanding and capture variations in responses.

## Results

### Participants

There were 13 participants in the demonstration for care providers: five physicians, seven ANMs and one *anganwadi* worker. Thirty-eight individuals participated in the demonstration for care receivers: 29 pregnant women and nine support persons. The support persons included three mothers, one mother-in-law, and five husbands. Of the 29 pregnant women, nine were from a private hospital, 10 from a UPHC, and 10 from an *anganwadi*; 12 of the 29 women were primiparous and the remainder were multiparous.

### Reflections on current ANC in Vadodara

Study participants were asked to share their perceptions of the strengths and challenges of the one-on-one model of ANC currently in place in Vadodara.

#### A typical ANC visit and goals for care

Participants were asked to describe a typical ANC visit and reflect on the goals for care during antenatal visits. Providers reported that a typical ANC visit includes confirmation of pregnancy (during the first ANC visit), lab tests, basic health measurements (weight, blood pressure, fundal height), and risk assessment to check for maternal and fetal wellbeing. In general, providers agreed that, in addition to the aforementioned physical exam and laboratory tests, ANC should include education on nutrition and self-care during pregnancy, and they emphasized the importance of attending all four recommended ANC visits. They reported that ANC services are typically provided by a physician with help from a staff nurse (in tertiary settings), an ANM or an accredited social health activist (ASHA) (in public facilities), or a second physician or medical assistant (in private hospitals). The schedule of visits is determined based on the woman’s gestational age upon entry into care; she is advised to return for her next visit at least once per trimester during her pregnancy. The public facility providers reported a range of 10–40 ANC cases per day, with higher volume on days when specialty care is available or when a dedicated obstetric clinic (such as Pradhan Mantri Surakshit Matritva Yojana, or PMSMY [17]) is being held. The private hospital providers reported 20–25 cases per day on average.

Pregnant women’s responses describing a typical ANC visit were congruous with those of providers, and mentioned physical exam, laboratory testing, as well as education on nutrition or recommended changes in activities of daily living. While women mentioned similar information and services that should be offered during each ANC visit as the providers did, many women also noted (*n* = 14) that assurance from the doctor was critical for wellbeing. Most pregnant women (*n* = 16) reported that ANC was provided either by a physician alone or a physician in combination with a staff nurse (*n* = 13). Pregnant women reported wait times ranging from 15 min to 4 hours. Women who had attended the ANC clinic at the tertiary hospital reported that the entire process (wait time plus visit) could take up to 6 hours. The majority of the pregnant women (*n* = 24) reported visiting more than one facility for ANC during their pregnancy: UPHCs were generally used to confirm the pregnancy and higher-level public facilities (hospitals) were used to address any complications. Women also noted that they would change facilities if they moved from their in-laws’ home to their natal home. In Vadodara, and India more broadly, many women adhere to the custom of *sava-mahina*: a woman experiencing her first pregnancy (although occasionally during subsequent pregnancies as well) will live primarily at her parents’ home and only return to her in-laws’ home 6 weeks after delivery. This custom can affect facility choice.

#### Strengths and challenges of the current model

Participants were asked about their views on the strengths of the current model of ANC in Vadodara. Providers cited outreach efforts that allowed them to provide access to care for more women, expanded service capacity (more facilities are able to do more advanced laboratory tests), the convenience that electronic health records in the urban primary health centers provide particularly for the management of high-risk pregnancies, and the provision of free services (e.g. delivery services, iron and calcium tablets) in public facilities. One physician remarked, “The current model is good in all ways, like we give tablets free of cost, all services are free of cost. Besides, we give all guidance based on the [MOHFW] guideline which is fully authorized and prepared by experts.”

When asked explicitly about any challenges perceived with the current model of ANC, the majority of providers (*n* = 8) and women (*n* = 26) did not report any challenges or frustrations. However, some providers noted frustration with a shortage of personnel at their facilities (*n* = 2) as well as problems with time management given the high volume of their caseloads (*n* = 3); this was noted as a problem particularly at the UPHCs where ANC is provided at the same time as the general outpatient clinic day (OPD). A provider from a UPHC stated, “I have to see 40 to 50 [OPD] patients and around 15 ANC [patients] in three and a half hours. [It is] …not only pregnant women—I have other patients also to whom I am not able to give sufficient time.” For their part, women expressed their dislike for the long wait times but also noted they were used to it.

### Reflections on the group ANC model

Following questions about the current model of ANC in Vadodara, participants were asked to share their reflections about what they had just seen and experienced during the group ANC demonstration.

#### Physical assessment

Participants were asked what they thought about the physical assessment component of the group ANC demonstration. Both providers and pregnant women were generally comfortable with all aspects of the physical assessment, including the time and attention given to each woman’s “belly check,” and having that exam done in a private area within the group space. Providers reported that they thought doing the clinical checkup in the group space worked well and would be feasible in their respective facilities. They also felt it was possible to maintain privacy and confidentiality despite being in a shared space. One physician and two ANMs noted they liked the low mat for women’s exams on the floor (versus an exam table) as it was easier for the pregnant women to get onto it. Regarding the checkup in the group space, one physician stated, “Yes, it is good, it is in the corner, so women don’t feel shy. All the patients are checked so they know that everybody is going to be checked, all enjoyed the process.”

The majority of women (*n* = 17) also agreed that the checkup in the group space worked well and was done in a way that maintained their privacy and confidentiality. One woman explained, “It was good, [the doctor] was talking all the time and I did not feel that… there was lots of noise in the room or that she was not able to listen to what I said. I did not feel anything like that. She talked softly yet I did not feel that I was in the same room [with everyone else].” However, three women, two of whom were from the private clinic and one from a public site, expressed discomfort with having their checkup done in the group space and stated that they would have preferred a private room.

#### Self-assessment

Participants were asked their opinions on the self-assessment components of the group ANC model. Overall, the self-assessment activities were well received by all participants. In general, providers felt that having the women take their own blood pressure (BP) and weight measurements was a novel idea, and helped women pay more attention and develop a feeling of ownership of their health information. One physician explained, “When [the women] measured their own weight they felt that they also can do something. There is empowerment of women. Because they had first time taken BP machine in hand. As we just taught them, they themselves used it so they felt happy from inside.” Another physician noted, “That was a great idea I think, because when we tell them that your BP is this much, we don’t know whether it registers with them or not. Here when they did [it] on their own, they notice their BP more. And peers are doing the same thing so they compare with them.”

The pregnant women also enjoyed taking their own BP and weight, noting that it made them pay closer attention to what the numbers meant and how those numbers can affect their health. According to one woman, “It was good we weighed ourselves and wrote in the report [worksheet] ourselves. We got to know how to check BP. I liked that I measured my BP on my own; in the hospital, we would not come to know [that information] as provider would just write it on their own. They do not tell us, so we do not realize.” The majority of the providers (*n* = 10) also felt that the self-assessment worksheets worked well. While they did express concerns that some women—particularly in less literate communities—may need assistance with completing the worksheets, they were confident that overall, women would get used to it and, when necessary, they could receive help. None of the women reported any difficulty or discomfort with the self-assessment worksheets used during the demonstration, even though a few had required assistance from their peers or the co-facilitator to complete the written worksheets.

#### Peer support and education

Participants were asked for their opinions about the learning component of the group ANC demonstration. Providers and pregnant women responded positively to the group discussion and group activities. The providers were highly enthusiastic about the peer support and education aspects of the model. They felt that group ANC could accomplish all of the goals of high-quality ANC while also providing more time for counseling and learning. Providers appreciated the interactive learning style that the discussion promoted. The physician who acted as a group leader for the demonstrations noted her surprise at how willing the women were to engage in the discussion: “Initially I was little skeptical whether I would be able to get answers [from the women]. But when we finally tried it, lot of information came from them, especially in the private [hospital] the level of knowledge was too good [meaning “very good”]. Even in the public [settings], in slums, though knowledge was not so much, we could encourage women to talk and many of the answers came from them.”

The women also reportedly enjoyed the discussion and stated that it gave them the opportunity to learn from the provider as well as each other. One pregnant woman explained, “Everyone gets chance to speak [and] due to that, a good atmosphere is also created. If only the service provider speaks, after sometime it feels like a lecture. As everyone speaks and shares their experience, we get to learn from that also. If I’m not suffering at present but some other lady is suffering and I hear her experiences, and after some time if I have a similar problem then I will remember about the discussion at that time.” Another pregnant woman noted that the group discussion was helpful for participants who were shy; although they did not speak up themselves, they had the chance to learn from others who might be going through similar situations.

Both providers and pregnant women also felt that the group activity was an effective, entertaining way to learn about information related to pregnancy (in the demonstration the topic was common discomforts and danger signs). Providers liked that the activity was fun and interactive while still being informative. One demonstrator remarked, “[This activity was a] good one. You learn when you are having fun. I didn’t find that women were getting bored or were in hurry, everyone was pretty attentive. I could see that they were happy playing games and learning. It is a better way of teaching.” The same demonstrator noted that she had been worried that women might copy one another’s answers or could be unwilling to share personal information about discomforts but that the women proved otherwise, offering their original thoughts and experiences.

The pregnant women also reported positive feedback on the group activity. One woman said that she preferred it to a one-on-one meeting with the physician: “If we all are together we do enjoy having fun (*masti ho jaati hai)* and we also learn. We like to learn more in the form of games rather than sitting alone, waiting for the doctor; that I don’t like.”

### Other aspects of the model

Participants were asked for feedback on other aspects of the model, including a set schedule of visits and the duration of each session. Five providers expressed concerns about introducing a set schedule of visits for group ANC. Three providers were worried about what would happen if they were called away to attend an emergency during a scheduled group sessions. They also expressed concern about women forgetting pre-scheduled sessions. Pregnant women, on the other hand, expressed a preference for the idea of a set schedule of appointments, and thought it would allow them to better manage their time. One woman explained: “That would be good that in advance we know the schedule, so we can finish other work and come.”

When asked for their opinions on the length of the group sessions (90–120 min), providers stated that it would be feasible and acceptable in each of their settings, although some thought it might present a challenge to manage OPD patients at the same time. Still, they reported that they would appreciate the increased efficiency of seeing 10–12 ANC patients in that period of time. However, providers from public facilities believed that 120 min would likely be necessary in settings serving less educated women in order to allow more time for discussion. A provider commented, “Initially again, I was doubtful that women will be ready to spend two hours, but we didn’t feel any time that any one of them was bored or was not attentive or was in a hurry to go home. In fact, I had an experience that they wanted to keep on talking. I didn’t feel that anyone felt like leaving even when session was over. I think if they have a good experience they would take out time.” And, “In a way we see, group ANC is good. Together for all 10 women, physical checkups are done and their BP, weight, etc. is completed in one and half hour to two hours. Otherwise in individual visit women come one by one so our lot of time goes in that. If we call women together, it will save our time, and they will get to know each other.”

Overall, pregnant women expressed satisfaction with the proposed session duration of 90–120 min because it was spent actively participating, rather than simply waiting to see the doctor. One woman remarked, “It is better to come over here rather than watching TV at home, at least by coming here we felt good and learnt something new.” In their focus group discussion, all of the husbands (*n* = 5) also agreed with that sentiment.

### Solutions to potential barriers

Participants were asked to identify any potential barriers to implementation in their setting, and to share their ideas about solutions that could help overcome those barriers. Providers made several concrete suggestions:

- Conduct sessions in the afternoons as most women are occupied with chores and household responsibilities in the mornings
- In addition to grouping women by similar gestational age, be sure to organize groups based on a common language (demonstration sessions showed that not all women attending a facility in Vadodara speak the same language)
- To ensure consistency of leadership, if one group leader is unable to join a session due to an unforeseen event, ensure that the co-facilitator, who is familiar with the group and its concerns, is available to lead the session
- To ensure continuity of care, if a pregnant woman is unable to join a session due to an unforeseen event, ensure that she can schedule an individual ANC visit so that her care is not interrupted
- Consider engaging an additional support person (not clinical staff) at the facility to manage the logistical aspects of the group (i.e. scheduling, pre-session reminders to the women)
- Engage leadership at a higher level in the (public) system to help support the implementing facilities and their staff

The providers from the public facilities felt that support from leadership at the health system level would be essential to the successful implementation of the group model. One ANM explained: “Yes, we can do [group ANC] but we need support for that. Meeting space for all the people, making groups [with a] minimum [of] 8–12 women.”

Three pregnant women felt that pre-session reminders (phone call or text) would help group ANC participants remember to attend each group visit, and one woman suggested that it might be helpful to have a physiotherapist join the sessions.

The potential barriers to implementing group ANC in Vadodara as well as the concrete solutions offered to overcome them, while hypothetical, provide useful insights and suggestions grounded in knowledge of an urban health system in the Indian context that should be tested through implementation research.

## Discussion

To our knowledge, this is the first study to evaluate the feasibility and acceptability of group ANC in India and the first study in Asia to evaluate a group model for pregnant women that includes a clinical care component. The findings provide evidence to support the rationale for future studies designed to evaluate the effects of group ANC on health outcomes, experiences of care, and system performance in India. The results of this qualitative assessment of stakeholders’ perceptions about the potential feasibility and acceptability of introducing group ANC in India were positive following participation in a demonstration of one mock session of the model.

While some participants expressed initial skepticism about the group model in concept, after participating in the demonstration the reaction of both providers and recipients of care was generally enthusiastic. Overall, pregnant women who participated were more open to the novel experience. Ultimately, both groups of participants saw group ANC as a vehicle for delivering more comprehensive ANC services, improving experiences of care, empowering women to become more active partners and participants in their care, and potentially addressing some current health system challenges. For example, both women and providers felt that group ANC might help to reduce wait times for women while care providers and administrators also expressed optimism that group ANC could improve patient retention in facilities. Care administrators identified patient retention as a priority goal as well as a challenge in Vadodara. The feedback gathered through the various qualitative methods also suggests some concrete system requirements that, if met, could help to ensure effective implementation of this model.

This study has some limitations. Due to resource and time constraints, we were only able to demonstrate one session of the full four-session model and participants’ responses and perceptions may have been different following a longer exposure to the model. Our study had a relatively small number of participants although the number was likely adequate given the qualitative methods used; however, we cannot fully ascertain the adequacy of the sample size as our study was not designed to test saturation. Our study instruments (focus group discussion guides, in-depth interview guides, and written survey) were not validated prior to use in this study. In addition, because the researchers were involved in the model demonstration as well as the data collection, there is a possibility of social desirability bias in the responses. Finally, because our study was conducted only in three urban facilities, the outcomes may not be transferable to other settings. Future research on group ANC in India could assess its feasibility and acceptability in a broader variety of settings, and evaluate outcomes of the model in both rural and urban facilities.

Our research complements and builds on the small but growing evidence base of studies assessing the feasibility of group ANC in LMICs. Similar to the findings of the three studies published to date that have collected stakeholder responses to group ANC [10–12], our research also recorded positive responses from study participants. The results of our study provide support for testing the group ANC model in urban India, as they indicate that the model would be both possible and welcome in this setting. We also gathered some specific suggestions to improve the feasibility of implementing group ANC in urban India. These include ensuring adequate staffing ratios to handle the volume and range of patients presenting to the clinics who are not enrolled in group ANC; hiring dedicated personnel to help with group ANC logistics and scheduling; giving attention to the space requirements for effective group ANC; and initiation of a system of appointment reminders for women. These insights could be useful to others planning to implement the model in similar LMIC settings.

This study is particularly timely given the recently updated WHO ANC recommendations (2016), which identify group ANC as a health systems intervention with the potential to improve the utilization and quality of ANC. The WHO report offers the following context-specific research recommendation:

“Group antenatal care provided by qualified health-care professionals may be offered as an alternative to individual antenatal care for pregnant women in the context of rigorous research, depending on a woman’s preferences and provided that the infrastructure and resources for delivery of group antenatal care are available” [1].

## Conclusions

This qualitative research study collected the perceptions of care providers, care administrators, pregnant women and their family support persons to assess the feasibility and acceptability of a model of group ANC that was developed through a systematic scoping review of group care in LMICs. It demonstrates positive responses from stakeholders of all types in a variety of care delivery settings following active participation in a demonstration of the model. It provides specific feedback from participants on each of the major components of the group care model: physical assessment, active learning, and peer support. It suggests concrete ways to facilitate the introduction of the group ANC model in urban India and to overcome potential system barriers to implementation.

## Abbreviations

ANC: Antenatal care
ANM: Auxiliary nurse midwife
ASHA: Accredited social health activist
BP: Blood pressure
CORT: Centre for operations research and training
LMICs: Low- and middle-income countries
MOHFW: Ministry of Health and Family Welfare
OPD: Out-patient clinic day
PMSMY: Pradhan mantri surakshit matritva yojana
PNC: Postnatal care
UPHC: Urban primary care clinic
WHO: World Health Organization
